# Research progress of sophoridine’s pharmacological activities and its molecular mechanism: an updated review

**DOI:** 10.3389/fphar.2023.1126636

**Published:** 2023-06-16

**Authors:** Yiwei Chen, Xiang Wang, Dongmei Ye, Zhousheng Yang, Qingrong Shen, Xiaoxia Liu, Chunxia Chen, Xiaoyu Chen

**Affiliations:** ^1^ Department of Pharmacy, Guangxi Academy of Medical Sciences and the People’s Hospital of Guangxi Zhuang Autonomous, Nanning, China; ^2^ School of Chinese Meteria Medica, Beijing University of Chinese Medicine, Beijing, China

**Keywords:** sophoridine, pharmacological activities, anti-tumor, natural product, molecular mechanism

## Abstract

**Background:** Sophoridine, the major active constituent of Sophora alopecuroides and its roots, is a bioactive alkaloid with a wide range of pharmacological effects, including antitumor, anti-inflammatory, antiviral, antibacterial, analgesic, cardioprotective, and immunoprotective activities. Sophora flavescens Aiton is a traditional Chinese medicine that is bitter and cold. Additionally, it also exhibits the effects of clearing heat, eliminating dampness, and expelling insects.

**Aims of the study:** To summarize the pharmacological research and associated mechanisms of sophoridine, we compiled this review by combining a huge body of relevant literature.

**Materials and methods:** The information related to this article was systematically collected from the scientific literature databases including PubMed, Google Scholar, Web of Science, Science Direct, Springer, China National Knowledge Infrastructure, published books, PhD and MS dissertations.

**Results:** Its antitumor activity is particularly remarkable, as it can inhibit cancer cell proliferation, invasion, and metastasis while inducing cell cycle arrest and apoptosis. Additionally, sophoridine also holds therapeutic potential for myocardial ischemia, osteoporosis, arrhythmias, and neurological disorders, primarily through the suppression of related inflammatory factors and cell apoptosis. However, sophoridine has also exhibited adverse effects such as hepatotoxicity and neurotoxicity. The antidisease effect and mechanism of sophoridine are diverse, so it has high research value.

**Conclusion:** As an important traditional Chinese medicine alkaloid, modern pharmacological studies have demonstrated that sophoridine has prominent bioactivities, especially on anti-tumor anti-inflammation activities, and cardiovascular system protection. These activities provide prospects for novel drug development for cancer and some chronic diseases. Nevertheless, the understanding of the multitarget network pharmacology, long-term *in vivo* toxicity, and clinical efficacy of sophoridine require further detailed research.

## 1 Introduction

**FIGURE 1:**
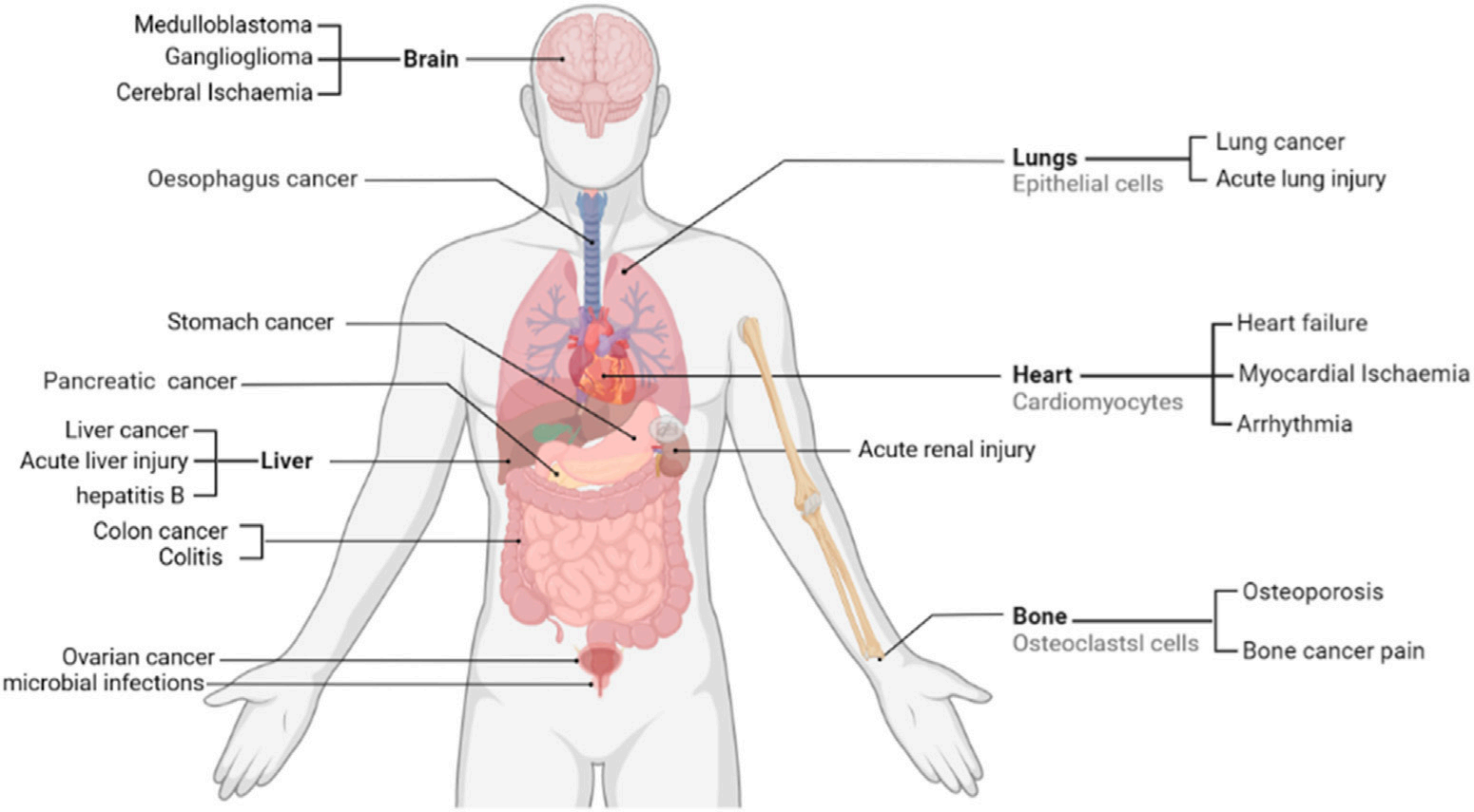
Therapeutic potential of sophoridine in humans. Current studies support that sophoridine may play a therapeutic role in the treatment of cancer, Cerebral Ischaemia, cardiovascular disease, and neurological effects.

Sophoridine (C_15_H_24_N_2_O) is a natural quinolone alkaloid mainly isolated from *Sophora alopecuroides* L. and *Euchresta japonica* Benth. More than 2 g of sophoridine can be extracted from 1 kg of *Sophora flavescens* (Zhao and Song, 2011; Shalaby et al., 2021). Sophoridine shows a variety of pharmacological activities, such as anti-tumor, anti-inflammation, antiviral, cardiovascular system protective, analgesic, and antibacterial effects (Chen et al., 2004; Zhang et al., 2006; Zhao et al., 2010; Yan and Wang, 2014; Quan et al., 2016a; Zhu et al., 2020). Following the discovery of its anti-tumor activity in 1977, sophoridine was approved for clinical research in 1993 (Li et al., 1987; Li et al., 2012). In 2005, the China Food and Drug Administration approved sophoridine for the treatment of various cancers, such as gastric, lung, and liver cancers, with low toxicity (Chen Y. et al., 2018). Additionally, sophoridine has good clinical prospects as a single-component drug; single-component medicine is advantageous for ensuring quality control compared with traditional Chinese medicine. Clinically, sophoridine is administered mainly through injection, enabling it to reach its site of action quickly through the bloodstream, with high bioavailability, has no first-pass effect, and the dosage is accurate. The various anti-disease effects and mechanisms utilised by sophoridine demonstrate its high research value. Table 1 summarises the basic molecular mechanism of sophoridine, a potential candidate for treating various diseases, such as cancer, inflammation, viruses, and cardiovascular diseases. The therapeutic potential of sophoridine in humans is shown in Figure 1. In this paper, the pharmacological action and mechanism of action of sophoridine are summarised to provide a reference for follow-up research. In contrast to the earlier review of sophoridine (Ur Rashid et al., 2020; Tang et al., 2022a; Wang et al., 2022), our work presents an extensive update on the pharmacological actions and molecular mechanisms underlying sophoridine’s effects, examines its pharmacological limitations, and suggests potential avenues for future research.

**TABLE 1:** Pharmacological activities of sophoridine.

Pharmacological effects	Activity/mechanisms of action	Cell lines/Model	Dose	Application	Ref
Anti-tumor activity	p53↓, FOXM1↓, CYR61↓, CDX2↓, VEGF↓, c-Myc↓	Lung cancer cells NCI-H446, NCI-H460, A549	5,10 μg/mL	*In vitro*	Xiong et al. (2017)
	CD86↑, F4↑, CD80↑	Lung cancer cells	20,40 μg/mL and 15,25 mg/kg	*In vitro/In vivo*	Zhao et al. (2021a)
	P53↑, mdm2↑, LATS−1↑, LAST2↑, YAP↓, CTGF↓	Lung cancer NCI-H460 cells	20 μg/mL and 16.9 mg/kg	*In vitro/In vivo*	Zhu et al. (2020)
	ROS↑, S phase↑, caspase-3/8↑, Survivin↓, Bcl-2↓, CDK-2↓, CD44↓, MMP2/9↓	Lung cancer A549 cells	5,10 μg/mL	*In vitro*	Li et al. (2015)
	MMP-2 ↓, MMP-9 ↓	Pancreatic Cancer capan-1 cells	1.25,2.5, 5 g/L	*In vitro*	Ren et al. (2017a)
	NF-κB P65↓, TNF-α↓, IL-1β↓, IL-6↓, IκB-α↑	Pancreatic Cancer capan-1 cells	0.625,2.5, 5 g/L	*In vitro*	Ren et al. (2016)
	G2 phase↑, Bax↑, bcl-2↓, pro-caspase-3↓	Pancreatic Cancer capan-1 cells	1,1.5, 2 g/L	*In vitro*	Ren et al. (2017b)
	Caspase-3 ↑	Pancreatic Cancer capan-1 cells	2.5,5 g/L	*In vitro*	Ren et al. (2017c)
	p-ERK↑, p-JNK↑, Blocked cells stay in the S phase, ROS↑	Pancreatic Cancer Miapaca-2 and PANC-1 cells	20μM and 20, 40 mg/kg	*In vitro/In vivo*	Xu et al. (2017a)
	M2-TAMs↓, M1-TAMs↓, M1-TAMs↓, CCR2↓, PD-1↓, TIM-3↓, LAG-3↓, TAMS↓	Gastric Cancer MFC cells	0.5,1,2 mg/mL	*In vitro*	Zhuang et al. (2020)
	HMGB3↓	Gastric Cancer MKN45 cells	2,3.5 mg/mL	*In vitro*	Chen et al. (2018b)
	S phase↑	Gastric Cancer cells MGC-803 cells	1.6 mg/mL	*In vitro*	Zhou et al. (2003)
	ESRRG↑, β-catenin↓, G2/M phase↑	Gastric Cancer AGS and SGC7901 cells	3 μM	*In vitro*	Peng et al. (2020)
	G0-G1 phase↑, S phase↑	Colon Cancer SW620 cells	2.8 mmol/L	*In vitro*	Liang and Zhang (2008)
	caspase-9↓, caspase-3↓, caspase-7↓, PARP↑	Colon Cancer SW480 cells	0.8 mg/mL and15,25 mg/kg	*In vitro/In vivo*	Liang et al. (2012a)
	MAPKAPK2↓	Colon Cancer HCT116, RKO, and SW480 cells	80,160 μM	*In vitro*	Wang et al. (2019)
	p53↓, VEGF↓	Colon Cancer SW480 cells	16.9 mg/kg	*In vivo*	Wang et al. (2010)
	ROS ↑, GSH ↓, Survivin↓, Livin↓, Bcl-2↓, E2F1↓, p27↓, CDK-2↓, caspase- 3/8↑, p53↑, Smac↑, Blocked cells stay in the G2/M phase	brain cancer U87MG cells	0.5, 1 mg/mL	*In vitro*	Wang et al. (2017b)
	FoxM1↓, NF-κB↓, AP-1↓	brain cancer U87MG cells	0.5, 1, 2 mg/mL	*In vitro*	Tsukamoto and Yokosawa (2006)
	FoXM1↓, TrkB↓, BDNF↓, NF-κB↓, AP-1, caspase-3/8↑	Brain cancer D283-Med cells	0.5, 1, 2 mg/mL	*In vitro*	Quan et al. (2013)
	caspase-3↑, DNA TOPI↓, EGFR-TK↓, APN↓, MMP-2 ↓	Brain cancer glioma U87 cells	5, 50, 100 μM	*In vitro*	Zhao et al. (2016)
	PTEN↑, Caspase-3↑, Caspase-9↑, PI3K↓, AKT↓, MMP-2↓, MMP-9↓	liver cancer HepG2 cells	5, 10, 20 μg/mL	*In vitro/In vivo*	Wang et al. (2017a)
	VEGFR2↓, RAS↓, MEK↓, ERK↓, resistance of liver cancer cells to lenvatinib↓	liver cancer HepG2 and Huh7 cells	20, 40, 80 μM	*In vitro/In vivo*	Zhao et al. (2021b)
	G0/G1 phase↑, FoxM1↓	esophagus cancer OE-19 and SK-GT2 cells	0.5, 1 mg/mL	*In vitro*	Chen et al. (2017)
	FHIT↑, PTEN↑, survivin↓	Ovarian cancer	25 mg/d	*In vitro*	Kou et al. (2016)
Anti-inflammatory	p-p38↓, iNOS↓, NO↓	RAW264.7 cellular inflammation model	15.63 mg/L	*In vitro/In vivo*	(Han et al., 2011)
	p38↓, iNOS↓, CD14↓, p-p38↓	RAW264.7 cellular inflammation model	15.63 mg/L	*In vivo*	Zhang et al. (2015)
	TNF-α↓, PGE_2_↓, IL-8↓	HL-60 cellular inflammation model	12.15, 48.6 mg/kg	*In vivo*	Huang et al. (2014)
	IL-6↓, TNF-α↓	IEC- 6 cellular inflammation model	50, 100 μM	*In vitro*	Zheng et al. (2014)
	P38↓, p-p38↓, NO↓	RAW264.7 cellular inflammation model	3, 6, 12 mg/kg	*In vivo*	Zhang et al. (2010)
	TLR-4↓, JNK↓, c-jun mRNA↓, c-jun ↓	RAW264.7 cellular inflammation model	31.25 mg/L	*In vitro*	Liu et al. (2016a)
	TLR4↓, c-Jun ↓, TNF-α↓, IL-1β↓	RAW264.7 cellular inflammation model	31.25 mg/L	*In vitro*	Liu et al. (2015)
	TLR4↓, NF-κB↓, TNF-α↓	RAW264.7 cellular inflammation model	31.25 mg/L	*In vitro*	Wang et al. (2011)
	TLR4↓, JNK↓, TNF-α↓	RAW264.7 cellular inflammation model	31.25 mg/L	*In vitro*	Wang et al. (2012)
	CD14↓, TLR4↓, TNF -α↓	Liver injury mice model	4, 6, 12 mg/kg	*In vivo*	Wang et al. (2009)
	PERK↓, TNF-α↓	Liver injury mice model	3, 6, 12 mg/kg	*In vivo*	Gao et al. (2010)
	IKKβ↓, NF-κB P65↓, TNF-α↓	Renal injury mice model	3,6,12 mg/kg	*In vivo*	Huang et al. (2011)
	IL-6↓,IL-10↓,NO↓,MDA↓, SOD↑	Lung injury mice model	2.5, 5 mg/kg	*In vivo*	Yang et al. (2012)
	TNF-α↓,IL-6↓	Lung injury mice model	5 mg/kg	*In vivo*	Han et al. (2006)
	SOD↑, MDA↓, NF-κB p65↓, NF-κB↓	Lung injury mice model	2.5, 5, 9 mg/kg	*In vivo*	Zhu et al. (2011)
	IL-6↓, MDA↓, NO↓, SOD↑	Lung injury mice model	2.5, 5, 9 mg/kg	*In vivo*	Tian et al. (2011)
	p-p38MAPK↓, c-jun↓, c-fos mRNA↓,TNF-α↓	Lung injury mice model	3, 6, 12 mg/kg	*In vivo*	Liang et al. (2012b)
	TLR4↓, MyD88↓, NF-κB↓, mTOR↓	Lung injury mice model	12 mg/kg	*In vivo*	Liang et al. (2022)
	ICAM-1↓, Hp↓, SIgA↓	Colitis rat model	25, 50 mg/kg	*In vivo*	Zhao et al. (2010)
Antiviral effects	Inhibited EV71 virus penetration into cells	EV71 virus	31.25, 62.5, 125, 500 and 1000 μg/mL	*In vitro*	Ren et al. (2019)
	Inhibit the adsorption and replication of EV71 virus RNA	EV71 virus	31.25,62.5 μg/mL	*In vitro*	Ou et al. (2016)
	IFNA↑, IL-10↑, TNF-α↓	Coxsackievirus B3	20, 40 mg/kg	*In vivo*	Zhang et al. (2006)
	virus replication↓	Coxsackievirus B3	8 g/L	*In vitro*	Yang et al. (2003)
	myocardial enzyme↓, MDA↓, LDH↓	Coxsackievirus B3	15.6, 31.3, 62.5 mg/L	*In vitro*	Liu et al. (2006)
	IFN-α↑, HBsAg ↓, HBeAg↓, HBV DNA↓	HBV virus	0.2 mmol/L	*In vitro*	Liu et al. (2016b)
	p38 MAPK↓, TRAF6↓, ERK1↓, NLRP10↓, caspase-1↓	HBV virus	0.4mM–1.6 mM	*In vitro*	Nie et al. (2007)
	Inhibited HBV virus penetration into cells at a dose-dependent manner	HBV virus	10, 100, 1000 mmol/L	*In vitro*	Chen et al. (2016b)
	p-PI3K↓, p-Akt↓, p-p38↓	HSV-1 virus	0.1, 0.2, 0.4 mg/mL	*In vitro*	Tang et al. (2022b)
Antibacterial activity	MIC = 12.5 μg/mL	*escherichia coli*	12.5, 25, 100 μg/mL	*In vitro*	Liu et al. (2011)
	MIC = 25 μg/mL	*staphylococcus aureus*			
	MIC = 25 μg/mL	*bacillus subtilis*			
	MIC(*escherichia coli*) = 2 × 10^−2^ mol/L	*escherichia coli*, *Bacillus* gasoformans, (proteusbacillus vulgaris, *bacillus subtilis*, *staphylococcus* albus	1 × 10^−2^-5×10^−2^ mol/L	*In vitro*	Xia et al. (2001)
	MIC(*Bacillus* gasoformans) = 2 × 10^−2^ mol/L				
	MIC(proteusbacillus vulgaris) = 4 × 10^−2^ mol/L				
	MIC(*bacillus subtilis*) = 2 × 10^−2^ mol/L				
	MIC(*staphylococcus* albus) = 1 × 10^−2^ mol/L				
	MIC (*escherichia coli*) = 8 mg/mL	*escherichia coli*	1, 2, 4, 8, 16, 32, 64, 128 mg/mL	*In vitro*	Quan et al. (2016b)
	MIC(proteusbacillus vulgaris) = 8 mg/mL	proteusbacillus vulgaris			
	MIC(*pseudomonas aeruginosa*) = 8 mg/mL	*pseudomonas aeruginosa*			
	MIC(*staphylococcus* epidermidis) = 4 mg/mL	*staphylococcus* epidermidis			
Cerebral ischemia Protection	TRAF6↓, p-ERK1/2↑	Permanent middle cerebral artery occlusion (pMCAO) model	2.5, 5, 10 mg/kg	*In vivo*	Liu et al. (2012)
	TLR4 ↓, NF-κB↓	Permanent middle cerebral artery occlusion (pMCAO) model	2.5, 10 mg/kg	*In vivo*	Miao et al. (2013)
	MDA↓,SOD↑,CAT↑*staphylococcus* epidermidis	Permanent middle cerebral artery occlusion (pMCAO) model	2.5, 5, 10 mg/kg	*In vivo*	Xue et al. (2020)
	TNF-α↓				
	, IL-1β↓, IL-6↓				
	p-PI3K ↑, p-Akt↑	Permanent middle cerebral artery occlusion (pMCAO) model	2.5, 5, 10 mg/mL	*In vivo*	Xue et al. (2021)
	IL-6↓, TNF-α↓, ET↓, NO↑	Permanent middle cerebral artery occlusion (pMCAO) model	10, 50 mg/kg	*In vivo*	Chen et al. (2010a)
Analgesic	COX-2 ↓, VEGF ↓	Spinal cord of rats with bone cancer pain	25 mg/kg	*In vivo*	Yan et al. (2013)
	NR2B mRNA↓, nNOS mRNA↓	Spinal cord of rats with bone cancer pain	25 mg/kg	*In vivo*	Yan and Wang (2014)
	Pain inhibition rate (%) = 55.63	Ratcwrithing response model, Rat auricle swelling model	10, 20, 30 mg/kg	*In vivo*	Qian et al. (2012)
	Swelling inhibition rate (%) = 17.66				
	pain threshold of mice↑	Abdominal pain rat model	15, 20, 25 mg/kg	*In vivo*	Zhang et al. (2005)
	tie of tenderness reaction↑				
Cardiovascular system protection	SERCA2a↑, Ca^2+^↑	Chronic heart failure rat model	5, 10, 20 mg/kg	*In vivo*	Middlekauff et al. (2012)
	Increased calcium volume in the sarcoplasmic reticulum, IL-1β↓, IL-6↓	Chronic heart failure rat model	5, 10, 20 mg/kg	*In vivo*	Hu et al. (2014)
	SERCA2a↑				
	ameliorating cardiac Ca^2+^ induced Ca^2+^ transientstie of tenderness reaction↑	Chronic heart failure rat model	2.5, 5, 10 mg/kg	*In vivo*	Hu et al. (2016)
	DHPR↑				
	±dp/dtmax↑tie of tenderness reaction↑	Chronic heart failure rat model	5, 10, 20 mg/kg	*In vivo*	Lu et al. (2012a)
	LVEDP↓tie of tenderness reaction↑				
	HM/BM ↓				
	Alleviate myocardial ultrastructural damage				
	Narrow the area of ischemic myocardial infarction	Myocardial ischemia rats model	2.5, 5, 10 mg/kg	*In vivo*	Ding et al. (2009)
	SOD↓, GSH-PX↓, NO↓, MDA↓	Myocardial ischemia rats model	2.5, 5, 10 mg/kg	*In vivo*	Din et al. (2009), Din et al. (2010b)
	LVSP↓, ±dp/dt↓, LVEDP↓	ischemia rats model	2.5,5, 10 mg/kg	*In vivo*	Din et al. (2010a)
	±dp/dtmax↑, LVEDP↓, HM/BM↓	rat model of myocardial infarction	5, 10, 20 mg/kg	*In vivo*	Lu et al. (2012b)
	Block the hERG K^+^ channel	HEK293 cells	300 μM	*In vitro*	Zhao et al. (2009)
	Ito↓, IKr↓, APDs↑, CVs↓, HR↓	LCAL-induced or ISO-induced arrhythmia model; Aconitine-induced SD rat arrhythmia model	100μM; 50, 100 mg/kg	*In vitro/In vivo*	Song et al. (2023)
Other activities	ERK↓, c-Fos↓, NFATc1↓	Mice bone marrow macrophage	5, 10, 15 μg/mL and 15 mg/kg	*In vitro/In vivo*	Zhao et al. (2017)
		Female FVB mice			
	IC_50_(pine wood) = 0.453 μg/mL	nematodes Bursaphelenchu, Xylophilus, *Meloidogyne incognita*, Panagrellus redivivus, *Caenorhabditis elegans*	5, 10, 20, 25 μg/mL	*In vivo*	Wang et al. (2016)
	IC_50_(southern root-knot) = 0.371 μg/mL				
	IC_50_(beautiful hidden rod nematodes) = 1.173 μg/mL				
	Antagonize the immunosuppression of 5-FU on spleen	Spleen of rat	20, 30 mg/kg	*In vivo*	Liang et al. (2011)

## 2 Methods

To identify *in-vitro* and *in-vivo* studies we searched the databases from their respective inceptions to May 2023, using the following terms and their synonyms: sophoridine, kushen, Sophora flavescens, pharmacology, toxicity and cancer. We focused on the research literature on the pharmacological effects of sophoridine published in English or Chinese. In addition, the literature was screened to remove those with simple studies, poor logic, and low experimental reliability. All literature screening should be conducted in accordance with the guidelines. We evaluated the included literature according to the experimental methods used by Heinrich et al. (2020), and the specific criteria are shown in Table 2 (Heinrich et al., 2020).

**TABLE 2:** Exclusion criteria for pharmacological correlation studies (Heinrich et al., 2020).

Category	Key words	Specific approaches
Experimental methods	Anti-tumor	Ideally, a comparison of the effect between tumor and healthy cells (if available), especially when the effect is observed at high concentrations, should be provided. It is essential to clearly separate out anti-cancer research (in general *in vivo*) and research on cytotoxic pro-apoptotic effects. The relevance of a cell line must be justified. Also, make sure to distinguish between chemopreventive agents and anti-cancer agents, and use a proper reference
	*In vivo* studies	Ascertain that the baseline data are sufficiently robust and sound and use the 3Rs for best practice using animals
	Appropriate controls	Define positive and negative controls and use preferably standard drugs from clinics
	Statistics	Triplicates are the lowest number of data for statistics. And weather the statistical tools adequate for the experimental approach
	Evaluation And conclusions	Any conclusion relating to future research needs must be specific and based on the data reported

The information related to this article was systematically collected from the scientific literature databases including PubMed, Google Scholar, Web of Science, Science Direct, Springer, China National Knowledge Infrastructure, published books, PhD and MS dissertations.

## 3 Anti-tumor effects

**FIGURE 2:**
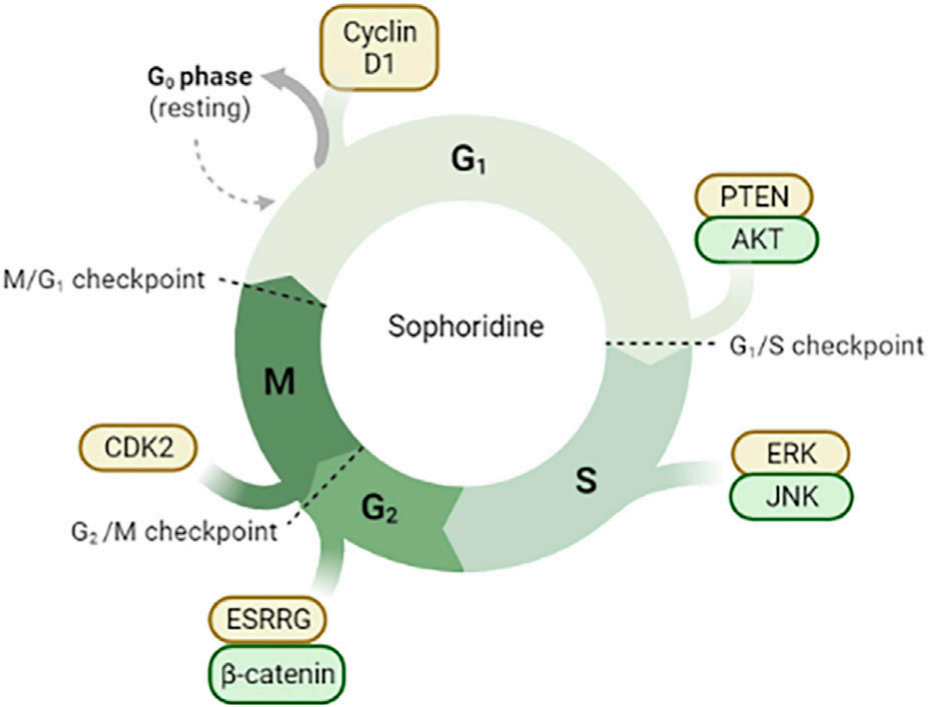
Sophoridine is involved in cell cycle arrest through different pathways. (1) Downregulation of Cyclin D1 protein expression in G0/G1 phase induces cell cycle arrest; (2) Inhibition of PTEN/AKT signal pathway in G1/S phase; (3) Inhibition of ERK/JNK signal pathway; (4) Inhibition of ESRRG/β-catenin signal pathway and downregulation of CDK2 protein expression.

Malignant tumors are among the most common and fatal diseases. In 2021, there were approximately 18 million new cancer cases and more than 8.7 million cancer-related deaths worldwide. The death toll is expected to increase to 13.1 million by 2030 (Miller et al., 2022; Siegel et al., 2022). Many medicinal plants can be used alone or in combination with commonly used chemotherapeutic drugs to prevent the occurrence and metastasis of cancer and further treat cancer (Cai et al., 2013; Ortiz et al., 2014). In 2005, sophoridine hydrochloride injection has been approved as an anticancer drug in China (Li et al., 2006). Sophoridine shows good anti-tumor effects in lung, pancreatic, gastric, colorectal, brain, and liver cancers *in vivo* and *in vitro* (Liang L. et al., 2012; Wang B. et al., 2017; Xu Z. et al., 2017; Yue et al., 2017; Peng et al., 2020; Zhu et al., 2020). Sophoridine inhibits proliferation and invasion and promotes apoptosis and autophagy of cancer cells. These effects are related to the inhibition of phosphatidylinositol 3 kinase (PI3K)/protein kinase B (AKT), Wnt/B-catenin, mitogen-activated protein kinase (MAPK)/extracellular signal-regulated kinase (ERK), and cell cycle pathways and promotion of the death receptor phosphatase and tensin homolog (PTEN) pathway. The anti-tumor effect and mechanism of sophoridine are described below according to the cancer type. As shown in Figure 2.

### 3.1 Lung cancer

Lung cancer is among the most common and fatal tumors. In 2022, approximately 350 people died of lung cancer each day in the United States. Non-small cell lung cancer accounts for 80% of all lung cancer cases. Currently, there is no effective treatment for lung cancer. (Bray et al., 2018; Siegel et al., 2022).

**FIGURE 3:**
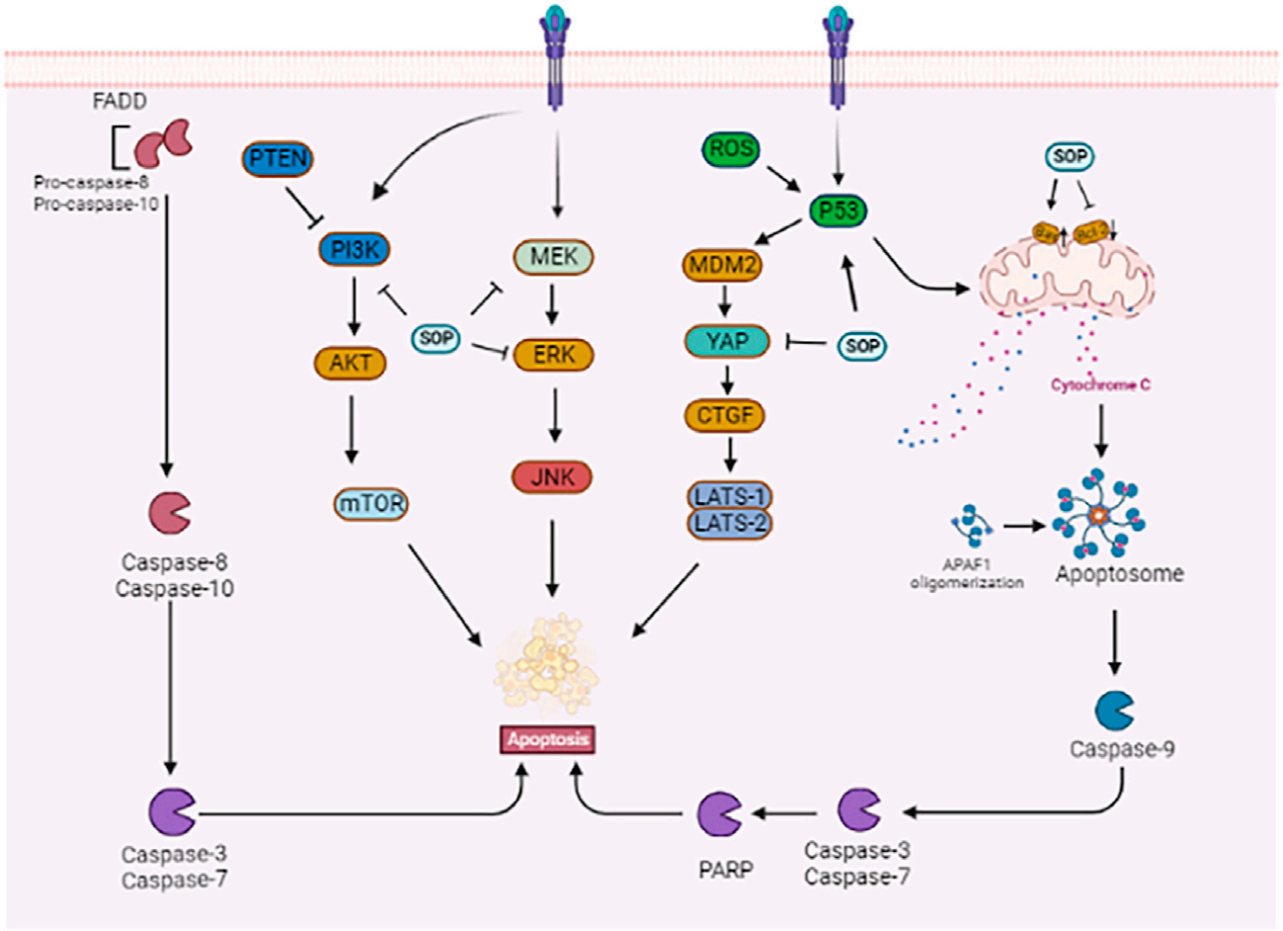
Related mechanism of sophoridine promoting apoptosis of cancer cells.

Zhao et al. showed that activation of the MAPK signalling pathway increased the expression of pro-inflammatory cytokines and macrophage M1 surface marker CD86, induced apoptosis of H460 and Lewis lung cancer cells, and inhibited cell clone formation and proliferation. In a Lewis-bearing mouse model, sophoridine (15 or 25 mg/kg) upregulated the expression of CD86/F4/80 in tumor tissues and significantly inhibited tumor growth (Zhao B. et al., 2021). Xiong et al. showed that sophoridine reduced cisplatin resistance in lung cancer cells and inhibited the proliferation of NCI-H446, NCI-H460, and A549 cells by activating the Hippo-YAP signalling pathway and p53 protein. Additionally, sophoridine significantly inhibited the expression of the target genes FOXM1, CYR61, CDX2, VEGF, and c-Myc downstream of the Hippo-YAP pathway (Xiong et al., 2017). Further studies showed that sophoridine enhanced the effect of cisplatin on lung cancer cells. Sophoridine increases the expression of p53 in a steady state by activating the Hippo and p53 signalling pathways and regulating p53 ubiquitination. Zhu et al. demonstrated *in vivo* that oral administration of sophoridine (16.9 mg/kg) for 4 weeks increased the expression of the p53, MDM2, LATS-1, and LAST2 proteins and significantly decreased the expression of YAP and CTGF in mice, thus inhibiting the proliferation, invasion, and migration of lung cancer cells (Zhu et al., 2020). Li et al. found that in A549 cells treated with different concentrations of sophoridine, the intracellular reactive oxygen species (ROS) level and apoptosis rate increased, and the cell cycle was blocked in the G2 phase. The expression of pro-apoptotic protein caspase-3/8 was upregulated, whereas that of apoptotic proteins survivin and Bcl2 and cell cycle-related protein CDK-2, adhesion molecule CD44, and matrix metalloproteinase (MMP)-2 and MMP-9 was downregulated. The results showed that sophoridine inhibited the proliferation and invasion of lung cancer cells by increasing ROS levels, promoting apoptosis, and blocking the cell cycle (Li et al., 2015). As shown in Figure 3.

### 3.2 Pancreatic cancer

The incidence and death toll of pancreatic tumors are increasing, with only 4% of patients surviving for 5 years after diagnosis. In addition, pancreatic cancer does not respond well to most existing chemotherapeutic drugs; therefore, studies are urgently needed to develop new anti-pancreatic cancer drugs (Vincent et al., 2011).

Sophoridine can kill pancreatic cancer cells but shows low cytotoxicity towards normal cells. ROS are necessary for sophoridine-induced cell cycle arrest and apoptosis. Sophoridine can also continuously activate the phosphorylation of ERK and c-Jun N-terminal kinase (JNK) and induce apoptosis of mitochondria-related cells through the JNK signalling pathway and cell cycle arrest in the S phase through the ERK signalling pathway (Xu Z. et al., 2017). In addition, sophoridine can activate the NF-κB signalling pathway; downregulate the expression of NF-κB p65, tumor necrosis factor (TNF)-α, interleukin (IL)-1β, and IL-6; and upregulate the expression of IκB-α. It further inhibits the proliferation and induces apoptosis of the pancreatic cancer cell line capan-1 (Ren et al., 2017a). Ren et al. showed that sophoridine (2.5 g/L) significantly downregulated MMP-2 and MMP-9 levels in capan-1 cells in a concentration-dependent manner (Ren et al., 2016). Sophoridine can also block capan-1 cells in the G2 phase, initiate a caspase cascade reaction, upregulate the expression of Bax, downregulate the expression of Bcl-2 and pro-caspase-3, induce apoptosis, and effectively inhibit the proliferation and invasion of pancreatic cancer (Ren et al., 2017b). Other studies showed that sophoridine increases the expression of caspase-3 and rate of apoptosis in capan-1 cells. When sophoridine (2.5 g/L) was combined with a caspase-3 inhibitor, the apoptosis rate of capan-1 cells decreased significantly, indicating that sophoridine also induces apoptosis of mitochondrial pathway cells through the caspase-3 pathway (Ren L. P. et al., 2017).

### 3.3 Gastric cancer

Because of its high incidence, short life cycle, and high mortality, gastric cancer remains the fifth leading cause of cancer-related death worldwide (Salati et al., 2019; Kozak et al., 2020).

The gene encoding HMGB3 is a novel oncogenic gene. Sophoridine (0.5–3.5 mg/mL) can downregulate HMGB3 expression, inhibit cell proliferation, and promote apoptosis in the gastric cancer cell line MKN45 (Chen X. D. et al., 2018). Sophoridine (0.4–3.2 mg/mL) acted on MGC-803 cells *in vitro*, resulting in pyknosis and nuclear chromatin aggregation. DNA electrophoresis revealed DNA “trapezoid” bands; the proportion of S phase cells increased according to flow cytometry, and sophoridine inhibited the growth of MGC-803 gastric cancer cells *in vitro* (Zhou et al., 2003).

**FIGURE 4:**
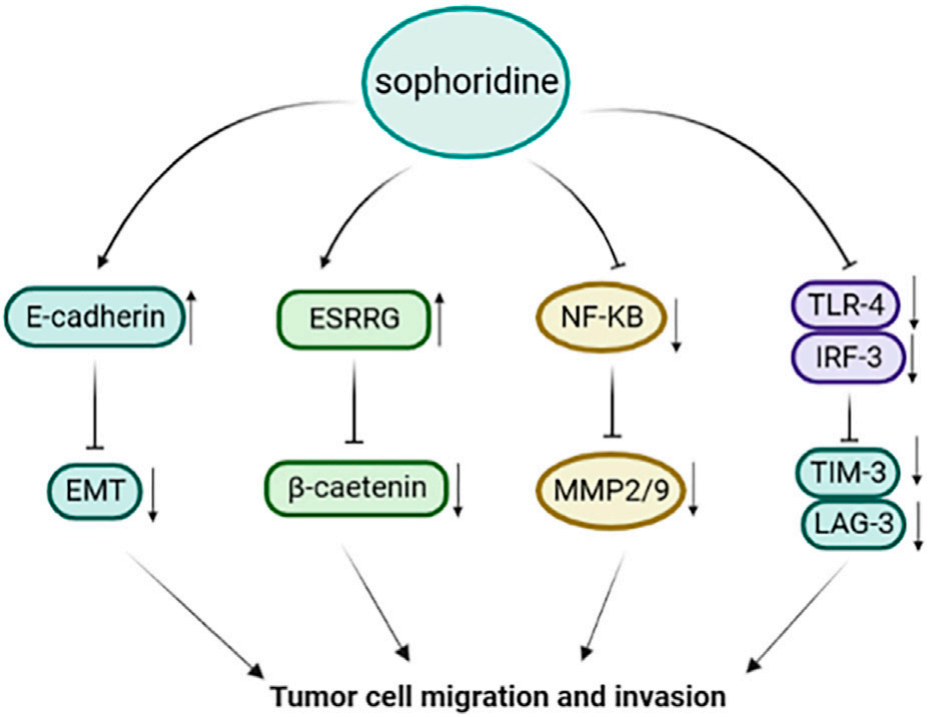
Sophoridine inhibits metastasis and invasion of cancer cells through multiple pathways.

Zhuang et al. found that sophoridine increased the polarisation of M1-tumor-associated macrophages through the Toll-like receptor 4 (TLR4)/interferon regulatory factor-3 pathway. Sophoridine downregulates the expression of C-C chemokine receptor type 2, cell failure markers programmed cell death protein-1, translocase of the inner membrane-3, and lymphocyte-activating 3 and inhibits the invasion of tumor-associated macrophages, thus enhancing the cytotoxic function of CD8+T cells and alleviating CD8+T cell failure (Zhuang et al., 2020). Peng et al. showed that sophoridine upregulated the expression of oestrogen-related receptor gamma, resulting in the degradation of β-catenin via a process not dependent on the ubiquitination-proteasome pathway, thereby inhibiting cell survival, invasion, and migration (Peng et al., 2020). In addition, sophoridine induced cell cycle arrest in the G2/M phase by inhibiting double-strand DNA break repair and enhanced the effect of cisplatin on gastric cancer cells. These findings provide preclinical evidence supporting sophoridine as a drug candidate. As shown in Figure 4.

### 3.4 Colon cancer

Human colon cancer is one of the most common malignant tumors and accounts for 10% of cancer-related deaths worldwide. Optimal treatment strategies for colorectal cancer are lacking. The combination of anti-epidermal growth factor receptor agents and chemotherapy is a treatment option for colorectal cancer but can cause serious toxic effects, seriously affecting the quality of life of patients (Siegel et al., 2014; Gharwan and Groninger, 2016).

Liang et al. showed that sophoridine acted on SW620 cells with a 48 h semi-inhibitory concentration (IC_50_) of 2.8 mmol/L. With prolonged sophoridine treatment, the proportion of S phase cells and number of apoptotic cells increased in a dose- and time-dependent manner (Liang and Zhang, 2008). In addition, sophoridine inhibited the growth of SW480 colorectal cancer cells in a time- and dose-dependent manner *in vivo* and *in vitro*. The IC_50_ of sophoridine was 0.78 mg/mL. The expression of caspase-9, caspase-3, and caspase-7 decreased, whereas that of PARP increased, thereby inducing the expression of apoptosis-related proteins (Liang L. et al., 2012). Wang et al. established a transplantable solid tumor using SW480 cells in nude mice and showed that sophoridine significantly inhibited the volume and mass of tumors by 34.07%. The expression levels of p53 and vascular endothelial growth factor (VEGF) in the sophoridine group were significantly lower than those in the control group. Thus, sophoridine may inhibit tumor growth by inhibiting the expression of p53 and VEGF (Wang et al., 2010). Wang et al. showed that the expression of MAPK-activated protein kinase 2 (MAPKAPK2) is closely related to the poor prognosis of colon cancer. Xase analysis of the PharmMapper and Kyoto Encyclopedia of Genes and Genomes databases revealed that MAPKAPK2 is a potential target of sophoridine. Western blotting showed that sophoridine significantly decreased MAPKAPK2 (Thr222) phosphorylation in a dose-dependent manner. Moreover, sophoridine directly binds to the ATP site of MAPKAPK2 according to molecular docking analysis. These results suggest that sophoridine can induce apoptosis and cell cycle arrest by targeting MAPKAPK2 and further inhibiting the occurrence of colorectal cancer (Wang et al., 2019).

### 3.5 Brain cancer

Brain tumors are primary and metastatic tumors of the central nervous system. In the United States, the annual incidence of primary malignant brain cancer is approximately 24,000, and approximately 70% of cases are highly invasive primary brain tumors. The survival time is only 15–16 months (Siegel et al., 2021; Siegel et al., 2022).

Sophoridine (1 mg/mL) activated the mitochondrial pathway to induce apoptosis in U87MG cells, upregulated the expression of survivin, livin, Bcl-2, and E2F1 and downregulated that of caspase-3/8, p53, and Smac. In addition, the expression of mitotic regulatory proteins p27 and CDK-2 decreased, and tumor cells were blocked in the G2/M phase. (Wang W. X. et al., 2017). In addition, sophoridine inhibited the ubiquitin-proteasome and transcriptional activities of Forkhead box M1 (FOXM1), NF-κB, and activator protein-1 in U87MG cells, which is consistent with the previous conclusion (Tsukamoto and Yokosawa, 2006). Based on these results, the mechanism of apoptosis in glioma cells induced by sophoridine may be related to the ubiquitin-proteasome pathway. Yue et al. found that sophoridine significantly inhibited the expression of FOXM1, tropomyosin receptor kinase B, brain-derived neurotrophic factor, NF-κB, and activator protein-1 and increased that of caspase-3/8 in D283-Med cells, a brain cancer-derived cell line. FOXM1 is a key regulator of the cell cycle (Quan et al., 2013). Studies showed that the expression of FOXM1 in D283-Med cells was inhibited. Sophoridine can inhibit the growth of human medulloblastoma cells by inhibiting the FOXM1, NF-κB, and activator protein-1 signalling pathways (Yue et al., 2017). Jiang et al. found that sophoridine inhibited the expression of the β-catenin protein and vimentin and MMP-9 mRNA and protein in human glioma U87 cells and enhanced that of E-cadherin. Sophoridine inhibits the migration and invasion of U87 cells. The mechanism may be related to the inhibition of the Wnt/β-catenin signalling pathway and blockage of epithelial-mesenchymal transition (Jiang et al., 2021). In addition, sophoridine may inhibit the invasion and proliferation of U87 cells by reducing the activities of DNA topoisomerase I, epidermal growth factor receptor-tyrosine kinase, amino-peptidase N1, and MMP-2; downregulating the NF-κB signalling pathway; and activating the apoptotic caspase-3 enzyme-linked reaction (Zhao et al., 2016).

### 3.6 Liver cancer

Treatment strategies for early liver cancer include hepatectomy, liver transplantation, and local ablation. However, most patients with liver cancer are diagnosed in an advanced stage, and the 5-year survival rate of patients with hepatocellular carcinoma is only 18% (Llovet et al., 2021). The morbidity and mortality of liver cancer in China rank fourth and third among cancers, respectively (Chen W. et al., 2016; Xia et al., 2022).

Wang et al. found that sophoridine treatment significantly inhibited the invasion and migration of hepatocellular carcinoma cells *in vitro*. The expression of PTEN, caspase-3, and caspase-9 proteins increased significantly, whereas that of PI3K, AKT, MMP-2, and MMP-9 proteins decreased in the sophoridine-treated group compared to that in the untreated group. In nude mice, the tumor volume and weight of the sophoridine-treated group decreased significantly in a dose-dependent manner compared to that of the control group. Sophoridine inhibited human hepatoma HepG2 cells by regulating the PTEN/PI3K/AKT, caspase-3, caspase-9, MMP-2, and MMP-9 signalling pathways (Wang B. et al., 2017). Lenvatinib is a newly approved multi-target tyrosine kinase inhibitor for the first-line treatment of advanced hepatocellular carcinoma. However, after long-term lenvatinib administration, liver cancer cells develop drug resistance, similar to the effects observed with other chemotherapeutic drugs. Zhao et al. found that sophoridine reduced the drug resistance of hepatocellular carcinoma cells to lenvatinib *in vitro*. In a nude mouse experiment, combined treatment with sophoridine and lenvatinib significantly decreased tumor volume, and sophoridine significantly reduced the resistance of hepatocellular carcinoma cells to lenvatinib. Western blotting showed that sophoridine further reduced the expression of VEGFR2 and downstream RAS/MAPK kinase/ERK in lenvatinib-resistant hepatocellular carcinoma cells by reducing the expression of ETS-1. The authors also revealed the potential mechanism of drug resistance of lenvatinib in hepatocellular carcinoma (Zhao Z. et al., 2021).

### 3.7 Other cancers

FoxM1 may be a target gene in tumor-targeted therapy and play an important role in tumorigenesis and progression. Chen et al. found that sophoridine significantly inhibited the proliferation of cardiac cancer cell lines OE-19 and SK-GT2 with the IC_50_ of 0.65 ± 0.09 and 1.14 ± 0.17 mg/mL at 72 h, respectively (Kalin et al., 2011; Wierstra, 2013). A double luciferase reporter gene assay showed that sophoridine significantly inhibited the transcriptional activity of the FoxM1 promoter and decreased the mRNA and protein expression of FoxM1. These data suggest that sophoridine can inhibit the proliferation of oesophageal-gastric junction adenocarcinoma cells *in vitro* by downregulating FoxM1 expression (Chen et al., 2017). Kou et al. divided 60 patients with ovarian cancer into control (cisplatin) and experimental (sophoridine and cisplatin) groups. The results showed that the expression levels of survivin were lower and those of fragile histidine triad protein and PTEN were higher in the experimental group than that in the control group. Sophoridine combined with cisplatin inhibits the proliferation of ovarian cancer cells. The mechanism may be related to increased expression of the tumor-suppressor genes fragile histidine triad protein and PTEN and decreased expression of the apoptosis inhibitor gene survivin. Sophoridine hydrochloride injection significantly inhibits tumors in nude mice. Using the DNA superhelical unspiral method, Ji et al. confirmed that the direct target of sophoridine hydrochloride injection was DNA topoisomerase I (Ji et al., 2006).

## 4 Anti-inflammatory effects

**FIGURE 5:**
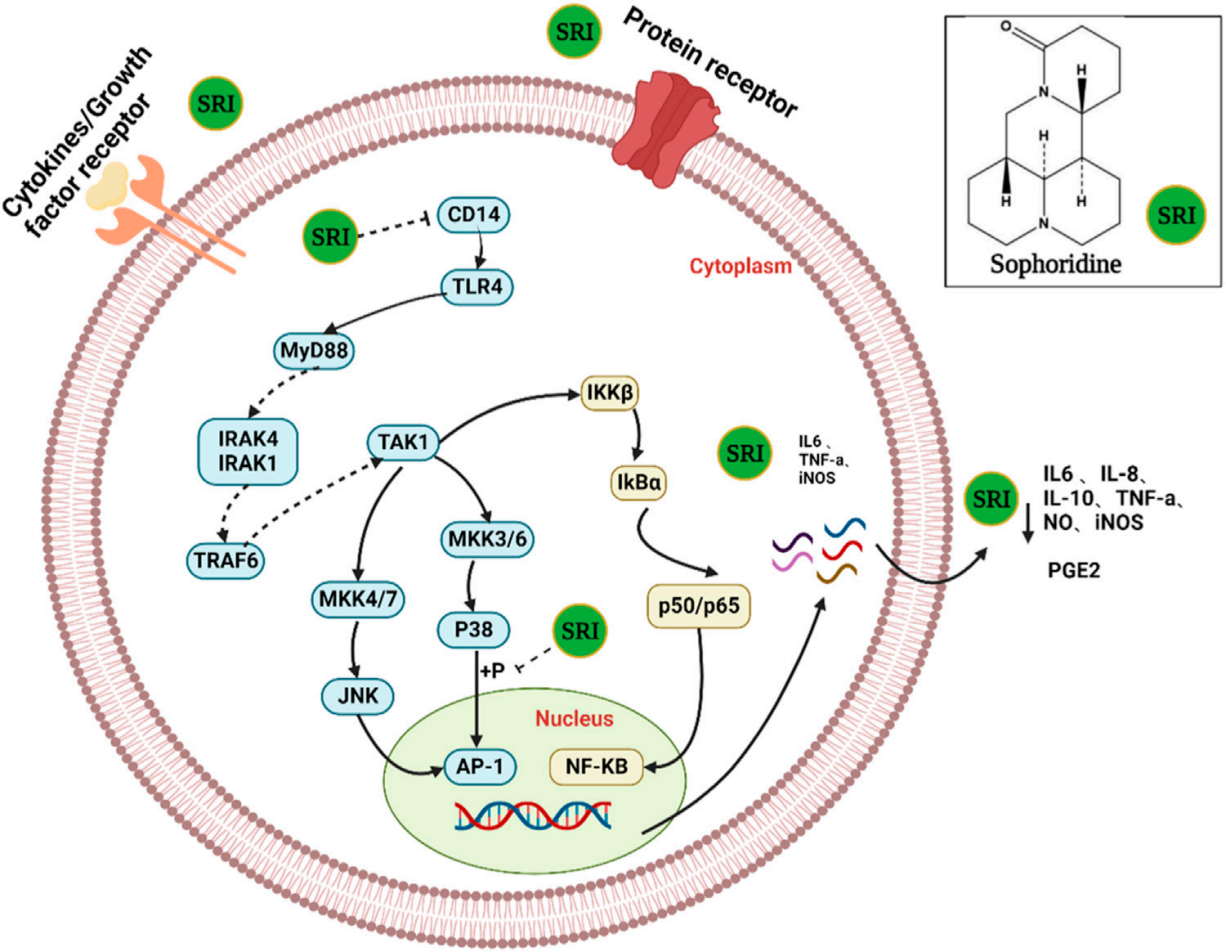
The possible mechanism of action of sophoridine in virus-infected cells. SRI can directly inactivate virus particles. More importantly, SRI may also suppress the activation of cellular PI3K/Akt and p38 MAPK pathways to reduce the subsequent replication of the virus and hence the production of virus progeny particles.

Sophoridine exerts anti-inflammatory effects by regulating the expression of inflammatory cytokines and chemokines TNF-α, IL-6, IL-8, IL-10; the pro-inflammatory transcription factor NF-κB; and inflammatory mediators. As shown in Figure 5. Sophoridine is used to treat gastroenteritis, acute lung injury, acute kidney injury, and hepatitis.

Huang et al. found that sophoridine inhibited the production of TNF-α, PGE2, and IL-8 in the cell supernatant from inflammatory models established *in vitro* (Huang et al., 2014). Sophoridine (100 μmol/L) also increased the survival rate of lipopolysaccharide (LPS)-injured cells by reducing the expression of IL-6 and TNF-α from 80.47% to 91.96% (Zheng et al., 2014). Liu et al. administered sophoridine via two methods to evaluate its effects on the expression of TLR4 and downstream JNK and c-Jun in LPS-activated RAW264.7 macrophages. The results showed that the expression of TLR4, JNK, and c-Jun mRNA and protein in the sophoridine group was significantly lower than that in the LPS group. Sophoridine exerts its anti-LPS effect by regulating the TLR4-JNK signal transduction pathway, and the effects observed using different administration methods suggest that its effect may be multiple links (Liu et al., 2015; Liu J. et al., 2016). Zhang et al. found that sophoridine downregulated the expression of p-p38 and inducible nitric oxide synthase (iNOS) proteins by inhibiting the p38MAPK site and nitric oxide (NO) release, exerting an anti-inflammatory effect (Zhang et al., 2010; Han et al., 2011). To further explore its anti-inflammatory pharmacological mechanism, Zhang et al. used two administration modes of sophostine (pre-treatment and pre-mixing) and showed that sophostine significantly downregulated the mRNA expression of p38 and iNOS and protein expression of CD14, p-p38, and iNOS in mouse RAW264.7 macrophages induced by LPS. The difference between the two administration methods was that the mixture of sophoridine and LPS downregulated CD14 mRNA expression in RAW264.7 macrophages induced by LPS, whereas sophoridine pre-treatment did not. Thus, sophoridine may exert anti-inflammatory effects by regulating the expression of CD14, p38, and iNOS (Wang et al., 2009; Zhang et al., 2015). In addition, Wang et al. confirmed that TLR4/myeloid differentiation factor-2 is one of the targets of sophoridine and that inhibiting activation of the TLR4/NF-κB/TNF-α pathway may be one of its anti-inflammatory mechanisms (Wang et al., 2011; Wang et al., 2012). In addition, the incidence and cancer rate of colitis is increasing rapidly worldwide as an important digestive tract disease (Ng et al., 2017). Zhao et al. found that sophoridine inhibited the expression of the intercellular adhesion molecule-1 gene, reduced plasma haptoglobin, and maintained caecal secretory IgA levels in dextran sulphate sodium-induced colitis in C57BL/6 mice. Thus, sophoridine may be an effective drug for treating inflammatory bowel diseases (Zhao et al., 2010).

Acute lung injury, a common, serious, and complex pulmonary inflammatory disease caused by a variety of pathogens and factors, is characterised by overexpression of inflammatory factors. Yang et al. found that sophoridine reduced the levels of IL-6, IL-10, NO, and malondialdehyde (MDA) in the serum of mice with acute lung injury induced by endotoxin (LPS), increased the content of superoxide dismutase, improved pathological injury of the lung, and enhanced the ability of antioxidant injury, ameliorating acute lung injury (Han et al., 2006; Tian et al., 2011; Yang et al., 2012). In addition, Zhu et al. found that sophoridine inhibited the expression of NF-κB, thereby blocking the TLR4-mediated NF-κB pathway in the LPS signal transduction pathway and alleviating lung injury (Zhu et al., 2011). Liang et al. found that sophoridine inhibited the phosphorylation of p38MAPK in the lung tissue of endotoxaemia mice, downregulated the expression of c-Jun and c-Fos mRNA, and inhibited the expression of the downstream inflammatory factor TNF-α (Liang J. P. et al., 2012). In addition, Liang et al. suggested that sophoridine inhibits LPS-induced acute lung injury by downregulating TLR4/MyD88/NF-κB and mTOR mRNA and protein expression in mouse lung tissue, further enhancing macrophage autophagy and reducing inflammation (Liang et al., 2022).

Huang et al. established a mouse model of acute renal injury induced by endotoxaemia. Sophoridine inhibited the expression of IKKβ and NF-κB P65 protein and TNF-α mRNA as well as nuclear translocation in the renal tissue and TNF-α expression in the serum (Huang et al., 2011). Gao et al. found that different concentrations of sophoridine significantly inhibited the expression of protein kinase RNA-like endoplasmic reticulum kinase and TNF-α in the liver to protect against acute liver injury (Gao et al., 2010). Wang et al. found that sophoridine (4, 6, and 12 mg/kg) inhibited liver inflammation and protected mice livers with endotoxaemia. The mechanism may be related to the downregulation of the expression of the LPS recognition receptors CD14 and TLR4 and inhibition of the secretion of downstream inflammatory factors (Wang et al., 2009).

## 5 Antibacterial and antiviral activities

Although great progress has been made in modern medicine, microbial infections remain a major challenge for health systems worldwide. Recent studies showed that sophoridine has an inhibitory effect on some microorganisms (Gasparini et al., 2012; Shallcross et al., 2015).

Xia et al. showed that the minimum inhibitory concentrations (MIC) of sophoridine against Escheri*chia coli*, *Bacillus aerogenes*, *Proteus*, *Bacillus subtilis*, and *Staphylococcus albicans* are 2 × 10^−2^, 2 × 10–^2^, and 4 × 10^−2^, 2 × 10^−2^, 1 × 10^−2^ mol/L, respectively (Xia et al., 2001). These results were confirmed in another study, which showed that sophoridine exerted significant antibacterial activity against *B. subtilis* and *Phytophthora infestans* (Liu et al., 2011). In addition, Quan et al. found that sophoridine inhibited *Pseudomonas aeruginosa* (MIC = 32.2 mM) and *Staphylococcus epidermidis* (MIC = 16.1 mM) in the genitourinary tract (Quan et al., 2016b). Other studies showed that sophoridine can inhibit the reproduction of the vaginal flora but does not destroy the normal physiological environment of the vagina (Wang et al., 1995).

**FIGURE 6:**
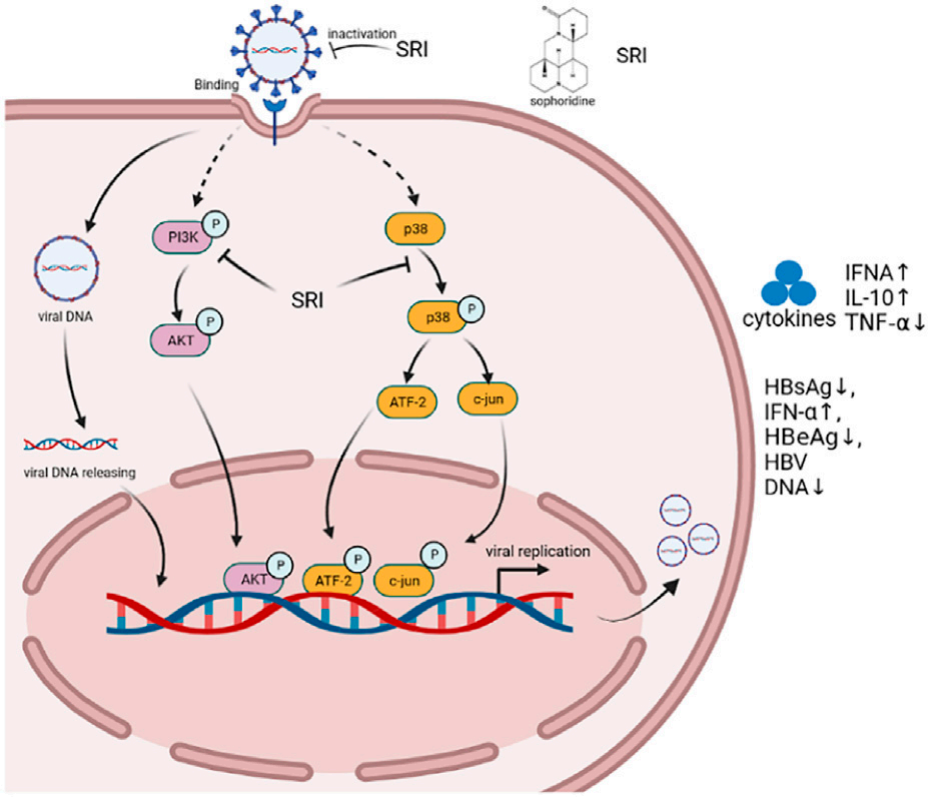
Schematic illustration of various Sophoridine-targeted inflammatory signaling pathways.

Sophoridine not only shows good antibacterial properties but also significantly inhibits viruses (Yang et al., 2003; Ou et al., 2016). Ren et al. compared the effects of sophoridine on enterovirus 71 induction in Vero cells before, during, and after viral adsorption. The results showed that sophoridine had an obvious antiviral effect on Vero cells treated with sophoridine before viral adsorption. The IC_50_ of sophoridine towards Vero cells was 354 μg/mL, and sophoridine (250 μg/mL) could protect 50% of cells from enterovirus 71 (Ren et al., 2019). Therefore, sophoridine is an effective drug against enterovirus 71 infection (Liu et al., 2006). In addition, sophoridine inhibited the cytopathic effect of Vero caused by coxsackievirus B3 infection. Further analysis showed that sophoridine combined with thymosin promoted the expression of interferon-α and inhibited the secretion of hepatitis B surface antigen and hepatitis B e-antigen as well as the replication of hepatitis virus DNA in HepG2 cells to exert antiviral effects (Liu X. Q. et al., 2016). Zhang et al. found that sophoridine had an obvious antiviral effect both *in vitro* and *in vivo*. Serum samples obtained from rats orally administered sophoridine revealed reduced viral titres in infected myocardial cells. Sophoridine significantly increased the mRNA expression of interferon-α and IL-10, decreased the mRNA expression of TNF-α, enhanced host resistance to viral infection, and inhibited cardiomyocyte apoptosis (Zhang et al., 2006). Chen et al. showed that the levels of p38 MAPK, TNF receptor-associated factor 6, ERK1, NOD-like receptor family pyrin domain containing 10, and caspase-1 decreased, and hepatitis B virus DNA methylation increased in HepG2.2.15 cells treated with sophoridine (0.4–1.6 mM) (Nie et al., 2007; Chen J. X. et al., 2016). Another study showed that 0.4 mM sophoridine inhibited 40.2% of hepatitis B surface antigen secretion, which was better than the effects of the positive control lamivudine (3TC, 31.5% at 1.0 mM) (Zhang et al., 2018). Further experiments showed that sophoridine downregulated the cellular PI3K/Akt signaling pathway and obstructed HSV-1 replication even more. Most importantly, SRI markedly repressed HSV-1-induced p38 MAPK pathway activation (Tang et al., 2022b). As shown in Figure 6.

## 6 Protective effect against cerebral ischaemia

Ischaemic cerebrovascular disease is a common and frequently occurring disease in the clinic. The mortality of ischaemic cerebrovascular disease is second only to that of heart disease (Feigin et al., 2016).

Sophoridine (10 mg/mL) reduces cerebral oedema and cerebral infarction volume, improves neurological deficits, and downregulates the expression of TLR4 and NF-κB. TLR4 and NF-κB may be novel targets for the treatment of cerebral ischaemia (Miao et al., 2013). Moreover, a high dose of sophoridine significantly reduces the levels of IL-6, TNF-α, and ET and increases the content of NO in the serum in a dose-dependent manner (Chen L. P. et al., 2010). In addition, Liu et al. measured the brain water content and infarct volume by establishing a proximal middle cerebral artery occlusion rat model treated with low-, medium-, and high-dose sophoridine. The results showed that compared with that in the control group, brain oedema of rats in the high-concentration sophoridine group (5 mg/kg) improved. In addition, in the low-concentration sophoridine group (2.5 mg/kg), the cerebral infarction volume decreased significantly, expression of TNF receptor-associated factor 6 decreased significantly, and phosphorylated ERK1/2 expression increased. Sophoridine can further protect the brain by regulating TNF receptor-associated factor 6 and ERK1/2 expression (Liu et al., 2012). In addition, sophoridine can reduce the MDA content in the brain of proximal middle cerebral artery occlusion rats, increase the activities of superoxide dismutase and catalase, and reduce the degree of oxidative stress in the brain tissue. Furthermore, sophoridine reduced the levels of the inflammatory cytokines TNF-α, IL-1β, and IL-6 in the serum, thus inhibiting the inflammatory reaction (Xue et al., 2020). Xue et al. further explored the mechanism and role of the PI3K/AKT signalling pathway in the anti-inflammatory and protective effects of sophoridine on cerebral ischaemic injury in rats. The results showed that sophoridine preconditioning significantly increased the levels of p-PI3K and p-AKT in ischaemic brain tissue but did not affect the expression of PI3K and AKT proteins (Xue et al., 2021). These studies support that sophoridine protects against cerebral ischaemic injury.

## 7 Analgesic effect

Approximately 20% of adults experience pain each year, among which 10% have chronic pain. Additionally, 20%–30% of people in China suffer from chronic pain (Goldberg and McGee, 2011). Sophoridine can relieve bone injury caused by tumors and increase the mechanical and thermal pain thresholds of bone cancer rats by downregulating the expression of cyclooxygenase-2 and VEGF (Yan et al., 2013). Sophoridine significantly downregulated the expression of N-methyl-D-aspartate receptor subtype 2B and neuronal NOS mRNA in the spinal cord of rats with bone cancer pain. Sophoridine may be involved in downregulating the N-meQian et al. studied the analgesic effect of sophoridine alkaloids by establishing acetic acid writhing pain, mouse ear swelling, celiac capillary permeability, and other inflammatory models. The results showed that sophoridine alkaloids had a significant analgesic effect and were superior to other alkaloids (Qian et al., 2012). Zhang et al. used a hot plate and tenderness test to verify the analgesic effects of sophoridine. In the two experiments, sophoridine at high, medium, and low doses significantly prolonged the pain threshold and reaction time to pain in mice (Zhang et al., 2005). Injection of *S. flavescens*, which contains sophoridine as one of its main components, is effective for treating middle and advanced malignant tumors and can effectively relieve pain in patients (Qi et al., 2013). In summary, sophoridine has a strong analgesic effect and should be further developed in the drug research and development fields to benefit patients with pain.

## 8 Cardiovascular system protection

### 8.1 Heart failure

Heart failure is a rapidly growing public health problem occurring in an estimated 37.7 million people worldwide, including 8.9 million patients in China; this rate is continuously increasing (Ziaeian and Fonarow, 2016; Group, 2022). Abnormalities in calcium transporters are observed in the skeletal muscle of patients with chronic heart failure, including in the heart tissue (Middlekauff et al., 2012). Matrine alkaloids, such as sophoridine, oxymatrine, sophocarpine, and matrine, with a common molecular structure of O=C=N-C-N, have positive inotropic effects on the myocardium (inversely regarded as negative inotropic action), possibly related to calcium channel activation (Chen et al., 2004). Hu et al. treated a rat model of chronic heart failure with medium and high doses (5 and 10 mg/kg) of sophoridine. SERCA2a protein expression in the sarcoplasmic reticulum of rat cardiomyocytes was significantly higher than that in the heart failure group. The results showed that calcium ion uptake increased, SERCA2a expression increased, the calcium capacity in the sarcoplasmic reticulum increased, calcium release required for the excitation-contraction coupling process increased, and myocardial contractility was enhanced, which was confirmed in a subsequent study (Hu et al., 2014). Sophoridine (5–10 mg/kg) can increase myocardial calcium-induced calcium transient and improve heart failure in rats, which is related to the upregulation of the dihydropyridine receptor. Moreover, compared with those in the heart failure group, the morphology of cardiomyocytes was significantly improved and mitochondrial ridges were closely arranged in the sophoridine-treatment group (Lu et al., 2012a; Hu et al., 2016).

### 8.2 Myocardial ischaemia

Damage caused by myocardial ischaemia is pivotal in diseases such as coronary heart disease and stroke, causing substantial mortality and morbidity (Burns et al., 2002; Lassen et al., 2013). Intravenous injection of sophoridine (2.5 and 10 mg/kg) can improve changes in left ventricular systolic pressure and ratio of the pressure change in the ventricular cavity during isovolumetric contraction period and reduce left ventricular end-diastolic pressure in rats with acute myocardial ischaemic injury (Din et al., 2010a). Sophoridine can protect the myocardium by ameliorating acute myocardial ischaemic injury. Further studies confirmed that sophoridine can dose-dependently reduce the size of myocardial infarctions caused by acute myocardial ischaemic injury (Ding et al., 2009). Ding et al. found that sophoridine increased the activity of superoxide dismutase and glutathione peroxidase and content of NO in the serum of rats with myocardial ischaemia, enhanced the function of the endogenous oxygen free radical scavenging system, significantly reduced the level of the serum lipid peroxidation product MDA, and reduced the damage of oxygen free radicals to the myocardium. Sophoridine scavenges free radicals and prevents lipid peroxidation, which may be one mechanism by which it protects cardiomyocytes from injury during ischaemia (Din et al., 2009). In addition, sophoridine protected against ultrastructural damage during acute myocardial ischaemia in rats. Compared with those in the model control group, the maximal rate of ventricular pressure increased and left ventricular end-diastolic pressure and heart mass/body mass decreased in the sophoridine-treatment group. Additionally, myofilament dissolution disappeared, and myofilaments were arranged neatly in the middle- and high-dose sophoridine groups (Din et al., 2010b; Lu et al., 2012b).

It has been reported that intravenous injection of sophoridine (2.5 and 10 mg/kg) can improve the changes of LVSP and ±DP/dt and reduce LVEDP in rats with acute myocardial ischemic injury. Sophoridine can protect the myocardium by improving acute myocardial ischemic injury. Further studies have confirmed that sophoridine can reduce the size of myocardial infarction caused by acute myocardial ischemic injury in a dose-dependent manner. Ding et al. found that sophoridine could increase the activity of SOD and GSH-PX and the content of NO in the serum of rats with myocardial ischemia, enhance the function of endogenous oxygen free radical scavenging system, significantly reduce the level of serum lipid peroxidation product MDA, and reduce the damage of oxygen free radicals to the myocardium. Sophoridine has the effects of scavenging free radicals and anti-lipid peroxidation, which may be one of the mechanisms of sophoridine in protecting cardiomyocytes from injury during ischemia. In addition, sophoridine also has a protective effect on ultrastructural damage during acute myocardial ischemia in rats. Compared with the model control group, ±DP/dtmax increased, LVEDP and HM/BM decreased in the sophoridine group, and myofilament dissolution disappeared and myofilament arranged neatly in the middle and high dose sophoridine group.

### 8.3 Arrhythmia

**FIGURE 7:**
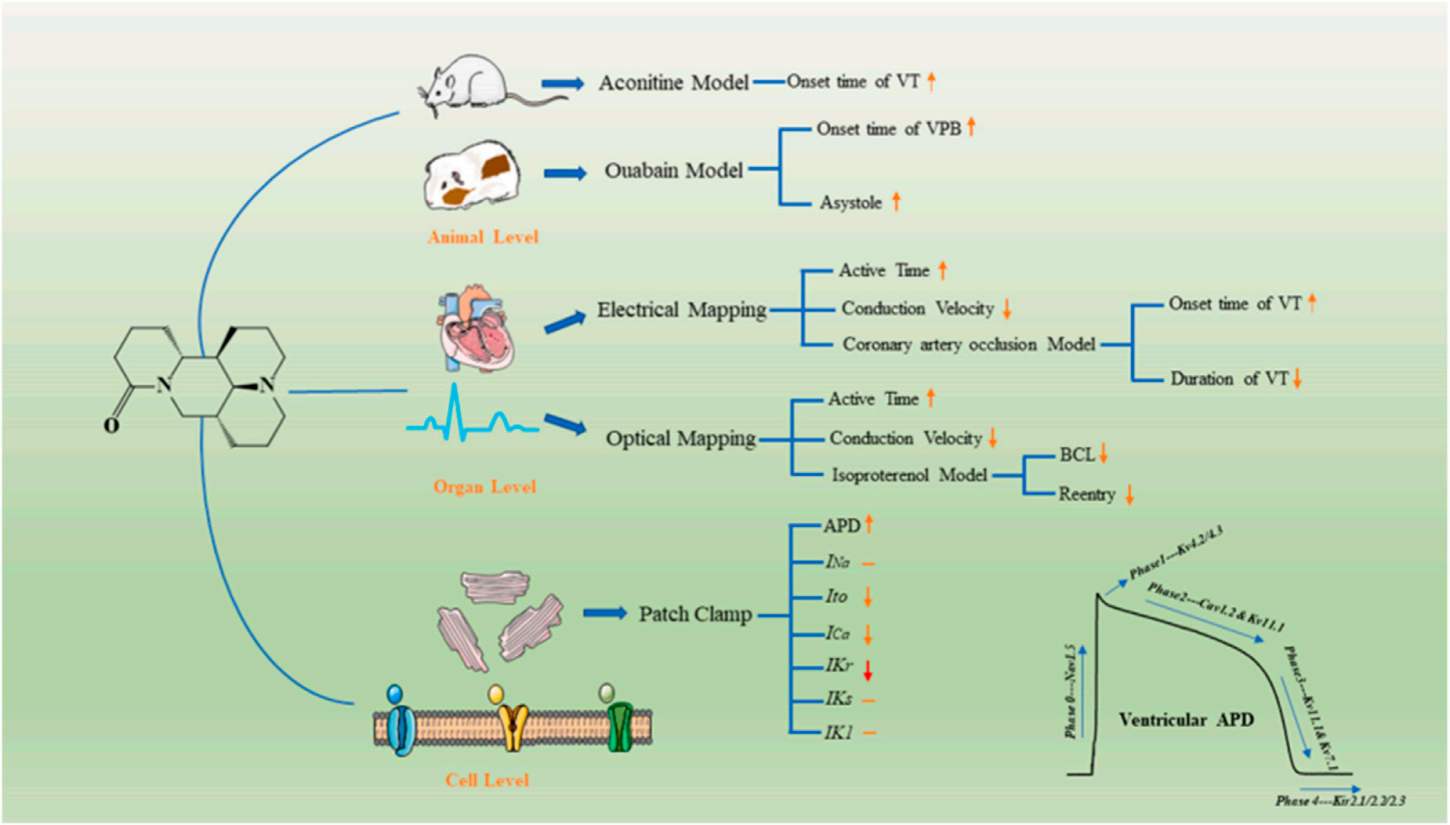
Illustration of electrophysiological effects and possible mechanisms underlying the antiarrhythmic effects of SR. hSC–CM: human-induced pluripotent stem cell-derived cardiomyocytes; MEA: microelectrode array; SR: sophoridine; FP: field potential; FPDc: corrected field potential duration[Ref (Song et al., 2023)].

Arrhythmia is a common cardiovascular disease. In China, 88% of sudden cardiac deaths are caused by malignant arrhythmias. The human ether-a-go-go-related gene (hERG) plays an important role in cardiac action potentials. Genetic mutations in the hERG gene can lead to an arrhythmic disease known as QT syndrome. Therefore, it is important to develop drugs that can block hERG channels (Sanguinetti and Tristani-Firouzi, 2006; Narayana Moorthy et al., 2013; Zhang et al., 2016). Sophoridine, an hERGK^+^ channel blocker with high binding affinity, changes the channel kinetics but does not affect the production and transport of hERG proteins (Zhao et al., 2009). In addition, according to the latest research, sophoridine manifested as a multiple ion-channel blocker in the electrophysiological properties and exerts antiarrhythmic effects *ex vivo* and *in vivo*. Meanwhile, due to the low pro-arrhythmic risk in the hERG inhibition assay and the induction of EAD, sophoridine has great potential as a leading candidate in the treatment of ventricular tachyarrhythmia. As shown in Figure 7.

## 9 Other pharmacological effects

Osteoclasts play key roles in osteoporosis development. Zhao et al. showed that sophoridine reduces the expression of NFATc1, the most important factor regulating osteoclast production, and inhibits osteoclast production by weakening RANKL-induced activation of ERK and c-Fos. Sophoridine exerted its anti-osteoporotic effect by inhibiting osteoclast formation (Zhao et al., 2017). The IC_50_ values of sophoridine against pine wood, southern root-knot, and beautiful hidden rod nematodes were 0.45, 0.37, and 0.78 μg/mL, respectively, which were significantly better than those in the positive control group (avermectin) (Wang et al., 2016). Liang et al. found that different doses of sophoridine had no significant inhibitory effect on the immune organs, thymus, and spleen, and sophoridine antagonised immunosuppression in the spleen caused by the first-line clinical antineoplastic drug, 5-fluorouracil (Liang et al., 2011).

## 10 Toxicity

In assessing a drug’s potency, it is crucial to evaluate its safety and toxicity, prioritizing these aspects. Numerous studies have been conducted over the past several decades to establish the safety and toxicity profile of sophoridine.

Liang et al. found that low doses of sophoridine affected mice’s appetite and weight, while higher doses caused severe symptoms (Liang et al., 2011b). Sophoridine also promotes rat liver BRL-3A cells apoptosis by increasing intracellular ROS accumulation. (Qiu et al., 2018). Additionally, sophoridine also exhibits neurotoxicity. Li et al. observed reversible neurotoxic reactions in rats after injecting sophoridine (32 mg/kg) for 60 days, but no histopathological changes were found (Li et al., 2004). In contrast, other studies reported that higher doses of sophoridine induced epilepsy in rats, with shorter latency and increased seizure success rate (Zhang et al., 2012). High doses of sophoridine can affect the hippocampus, causing persistent neuronal damage through the ERK pathway (Chen X. et al., 2010). Simultaneously, it can also lead to damage in rat hippocampal CA3 neurons, resulting in varying degrees of degeneration and endothelial cell damage (Chen X. et al., 2010). Furthermore, intraperitoneal injection of high-dose sophoridine can induce typical seizure-like behavior and epileptiform electroencephalogram in rats, leading to mitochondrial dysfunction in cells, upregulation of inflammatory factors such as TNF-α, IL-2, and IL-6, causing hindbrain damage, and triggering epilepsy (Chen et al., 2007). However, more animal models and clinical trials are needed to systematically evaluate toxicity. The toxicity of sophoridine is summarized in Table 3 for additional reference.

**TABLE 3:** Toxicity of sophoridine.

Activity/mechanisms of action	Model	Dosage/Route of administration	LD_50_(mg/kg)	Ref
Reducing autonomic activity and crouching	KM mice	40/50/62.5 mg/kg, ip	65.19	Liang et al. (2011b)
Neurotoxic reaction	SD rats	32 mg/kg, ip	—	Li et al. (2004)
reducing epilepsy incubation time and extends seizure duration	SD rats	47.83 mg/kg, ip	—	Zhang et al. (2012)
Activating the ERK signaling pathway; Up-regulating ERK1, ERK2 and p-ERK1/2 proteins	Epilepsy rat model	47.83 mg/kg, ip	—	Chen et al. (2010b)
In the hippocampal CA3 region, neuronal nuclear membrane bilayer structure is disrupted, neuronal nuclei exhibit severe shrinkage, and chromatin aggregates in a granular pattern	Epilepsy rat model	47.83 mg/kg, ip	—	Chen et al. (2010c)
Up-regulating TNF-α, IL-2 and IL-6	SD rats	55 mg/kg, ip	—	Chen et al. (2007)
Acute toxicity	KM mice	12.5/25/50 mg/kg, iv	50	Hu et al. (2012b)
Liver and kidney damage	KM mice	12/23/45 mg/kg, ip	62.6	Shi and Feng (2020)

## 11 Pharmacological activities of sophoridine derivatives

**FIGURE 8:**
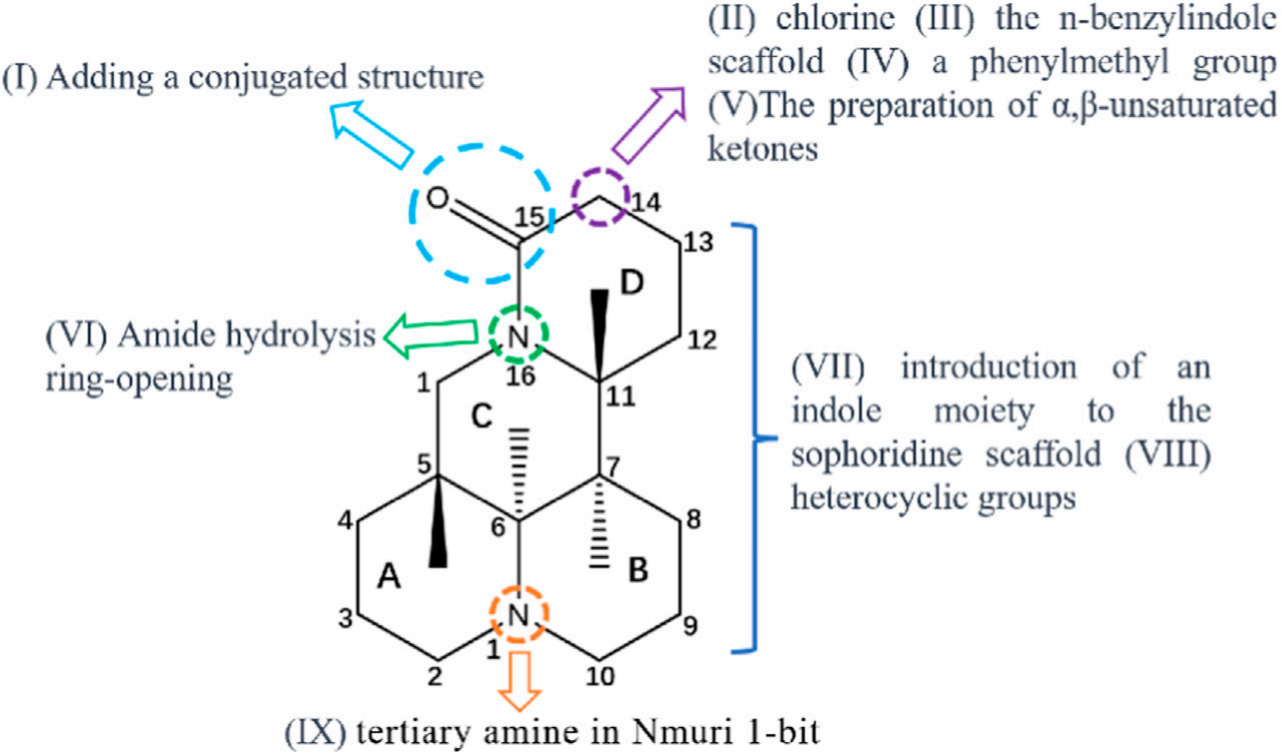
Structure activity relationship analysis of sophoridine and its derivatives.

At present, maintaining a balance between the therapeutic effect and toxicological safety of sophoridine remains difficult. To solve this problem, researchers have performed structural modifications to obtain many sophoridine derivatives; these derivatives have a significant therapeutic effect, low toxicity. Sophoridine has been modified as follows: (I) Adding a conjugated structure at the 15-carbonyl position through imine formation (Xu et al., 2018a); (II) Replacing the 14th position of sophoridine with chlorine (Gao and June 2013); (III) the n-benzylindole scaffold is combined with the C-14 atom of sophoridine (Li et al., 2018); (IV) Inserting a phenylmethyl roup at the C-14 position in the parent sophoridine metabolite (Tan et al., 2016); (V) preparation of α,β-unsaturated ketones (Xu Y. et al., 2017); (VI) Amide hydrolysis ring-opening (Bi et al., 2017); (VII) introduction of an indole moiety and heterocyclic groups to the sophoridinescaffold (Xu et al., 2018b); (VIII) tertiary amine in Nmuri 1-bit (Ni et al., 2017). The structure-activity relationship study of sophoridine and its derivatives is illustrated in the Figure 8.

In addition, many derivatives of sophoridine exhibit significant anti-tumor effects with reduced side effects, including myelosuppression. Sophoridine derivatives (38a-e) significantly inhibited the proliferation of S180 and H22 cells with IC_50_ values of 1–4 μM. Further studies showed that these metabolites inhibited the catalytic activity of topoisomerase I (TopoI) to prevent the binding of TopoI to DNA and inhibit DNA cleavage. The binding energies of these derivatives were similar to those of the classical TopoI inhibitors camptothecin and HPT (Liu K. et al., 2016). The sophoridine derivative 6b showed strong anti-tumor activity against three cancer cell lines (leukaemia K562, breast cancer human mammary epithelial cells, and HepG2) with IC_50_ values of 0.55–1.7 μM. Studies of the anti-tumor mechanism showed that metabolite 6b inhibited lysosome acidification, damaged lysosome function in cancer cells, and blocked autophagy flux, leading to tumor cell death (Bi et al., 2017). Dai et al. showed that sophoridine derivatives (8a–j) significantly increased autophagy flux, increased the expression of LC3-II and beclin-1, and decreased the level of p62, possibly by simultaneously inhibiting phosphorylation of p70S6K, 4E-BP1, and AKT (Dai and Wang, 2022). In addition, the tricyclic sophoridine derivatives 6b and imb-6g are autophagy inhibitors that induce autophagy dependence. Sophoridine 6b blocked autophagic flux in tumor cells and significantly inhibited lysosomal acidification, thus decreasing tumor cell survival. Lysosomal membrane permeabilisation induced by sophoridine ester imb-6g causes the release of cathepsins B and D lysosomes and eventually induces mitochondrial apoptosis. Autophagy is a biological process that promotes cell survival and induces death (Mirza-Aghazadeh-Attari et al., 2019). Inhibiting autophagy is a promising strategy for cancer treatment, as autophagy is upregulated in cancer cells treated with chemotherapeutic drugs (Wang Y. X. et al., 2017). However, the nature of autophagy remains unclear and thus requires further analysis (Jiang et al., 2019). Most studies were focused on improving the anti-tumor pharmacological activity of sophoridine through structural modification (benzyl indolyl or chlorophenyl) and opening of the lactam ring. However, other pharmacological activities (inflammation, viruses, and fibrosis) have not been extensively evaluated. Research in this area should be expanded to screen sophoridine derivatives with higher activity and fewer side effects.

## 12 Discussion and future prospect

Natural product therapy has been widely studied as an alternative treatment for cancer (Lefranc et al., 2019; Robinson et al., 2019). The structures and anti-tumor mechanisms of many natural products have been determined. Because of their multiple advantages, including diverse structures, multiple targets, high activity, and low toxicity, natural products may be valuable resources as multi-target drugs (Sheng and Sun, 2011; Qiao and Zhang, 2014).

Sophoridine is a natural, multi-target anti-tumor molecule. The current pharmacological research focus of sophoridine worldwide is on anti-tumor effects. It exerts its anti-tumor activity through numerous molecular mechanisms, such as the caspase-dependent, ROS-dependent, MAPK/ERK, PI3K/AKT/mTOR, NF-κB, and Hippo/YAP activation signalling pathways (Wang B. et al., 2017; Xu Z. et al., 2017; Peng et al., 2020). Interestingly, sophoridine can not only inhibit the proliferation and induce the apoptosis of cancer cells but can also maintain cell proliferation and inhibit the apoptosis of ordinary cells in a pathological state.

NF-κB is an important cellular nuclear transcription factor involved in the inflammatory response, regulation of apoptosis and stress response, and overactivation of NF-κB (Dolcet et al., 2005). Sophoridine inhibits tumor cell proliferation and induces apoptosis by inhibiting NF-κB. However, when lung epithelial cells are under stress, sophoridine downregulates the expression of NF-κB, inhibits cell apoptosis, and maintains the survival of lung cells (Zhu et al., 2011). The Hippo/YAP pathway is associated with cell proliferation, tissue homeostasis and tumorigenesis (Yu et al., 2015; Moya and Halder, 2019). Sophoridine inhibits lung cancer cell growth and enhances cisplatin sensitivity by activating the p53 and Hippo signaling pathways (Xiong et al., 2017). The regulation of oxidative stress is an important factor in both tumor development and responses to anticancer therapies. Many signalling pathways that are linked to tumorigenesis can also regulate the metabolism of reactive oxygen species (ROS) through direct or indirect mechanisms (Gorrini et al., 2013). Sophoridine induces tumor cells to produce ROS, activates the caspase-dependent mitochondrial pathway, leads to oxidative stress, and further induces endogenous apoptosis (Xiong et al., 2017). At the same time, in ischemic disease models, sophoridine inhibits tissue oxidative stress, reduces inflammation factor levels, suppresses ROS production, and consequently inhibits the apoptosis of neuronal and myocardial cells (Xue et al., 2020). Curcumin, tanshinone, and artemisinin exhibit similar killing and protective effects on cells (Lu et al., 2008; Feng et al., 2017; Wu et al., 2018; Maeda et al., 2019). Disorders in the PI3K/AKT signalling pathway occur in a variety of human diseases, including cancer, cardiovascular disease, and neurological diseases. PI3K is an important coordinating factor in the intracellular signalling response to extracellular stimuli; it can alter the protein structure of AKT, activate AKT, and activate or inhibit a series of downstream substrates by phosphorylation, such as apoptosis-related protein caspase-9 activity, thus regulating cell proliferation, differentiation, apoptosis, and migration (Saeed et al., 2019). Sophoridine inhibits the PI3K/AKT pathway in hepatocellular carcinoma cells but not in normal cells (Wang B. et al., 2017; Xue et al., 2021). MAPK/ERK regulates various cellular activities, including proliferation, differentiation, apoptosis, survival, and inflammation. Sophoridine activates the MAPK/ERK signaling pathway, promoting the phosphorylation of ERK1/2 and JNK, significantly inhibiting the proliferation of cancer cells. Moreover, sophoridine can also suppress inflammation factors related to cerebral ischemia and osteoporosis through the MAPK/ERK signaling pathway, further alleviating symptoms. In summary, sophoridine exhibits a wide range of pharmacological activities, among which its anti-tumor mechanism is closely associated with multiple pathways such as NF-κB, Hippo/YAP, oxidative stress, PI3K/AKT, and MAPK/ERK. However, other effects of sophoridine, such as inducing cell differentiation and immunomodulation, should be further evaluated.

In terms of experimental research, the high-dose usage of sophoridine is an urgent issue that needs to be addressed, as it may raise questions about the scientific validity of the research results. In most *in vitro* cell experiments, there is a lack of control groups consisting of normal healthy cells, leading to one-sidedness in the experimental outcomes. Particularly in animal studies, the daily dosage is typically calculated based on the animal’s weight, but this method has its limitations. In mouse models, due to their fast metabolism, the administered dosage often exceeds several times the standard clinical dosage. Additionally, different research teams employ varied administration methods and dosages, which can potentially yield misleading experimental results. Presently, investigations into sophoridine toxicity mainly center on hepatotoxicity, nephrotoxicity, and neurotoxicity, while research on other organs remains scarce. In the realm of reproductive toxicity, we note that the current literature is both limited and outdated, lacking compelling evidence. Consequently, we advocate for further in-depth exploration of the additional toxicities associated with sophoridine, with particular emphasis on reproductive toxicity. To address these issues, researchers should adhere to a unified and authoritative guideline (Heinrich et al., 2020) for conducting *in vivo* or *in vitro* studies on the pharmacology and toxicology of sophoridine. Only by doing so can we provide better evidence for its clinical application.

In general, sophoridine exhibits a wide range of pharmacological effects and shows promising therapeutic potential for various diseases. However, existing studies also have certain limitations. Therefore, it is crucial to employ rigorous scientific methods in the research of sophoridine.
